# Sequential gene promoter methylation during HPV-induced cervical carcinogenesis

**DOI:** 10.1038/sj.bjc.6604055

**Published:** 2007-10-30

**Authors:** F E Henken, S M Wilting, R M Overmeer, J G I van Rietschoten, A O H Nygren, A Errami, J P Schouten, C J L M Meijer, P J F Snijders, R D M Steenbergen

**Affiliations:** 1 Department of Pathology, Unit of Molecular Pathology, VU University Medical Center, Amsterdam, The Netherlands; 2 Department of Molecular Cell Biology & Immunology, VU University, Amsterdam, The Netherlands; 3 MRC-Holland, Amsterdam, The Netherlands

**Keywords:** MS-MLPA, Ms-SNuPE, MSP, DNA methylation, cervical cancer, transformation, biomarker

## Abstract

We aimed to link DNA methylation events occurring in cervical carcinomas to distinct stages of HPV-induced transformation. Methylation specific-multiplex ligation-dependent probe amplification (MS-MLPA) analysis of cervical carcinomas revealed promoter methylation of 12 out of 29 tumour suppressor genes analysed, with *MGMT* being most frequently methylated (92%). Subsequently, consecutive stages of HPV16/18-transfected keratinocytes (*n*=11), ranging from pre-immortal to anchorage-independent phenotypes, were analysed by MS-MLPA. Whereas no methylation was evident in pre-immortal cells, progression to anchorage independence was associated with an accumulation of frequent methylation events involving five genes, all of which were also methylated in cervical carcinomas. *TP73* and *ESR1* methylation became manifest in early immortal cells followed by *RARβ* and *DAPK1* methylation in late immortal passages. Complementary methylation of *MGMT* was related to anchorage independence. Analysis of nine cervical cancer cell lines, representing the tumorigenic phenotype, revealed in addition to these five genes frequent methylation of *CADM1*, *CDH13* and *CHFR*. In conclusion, eight recurrent methylation events in cervical carcinomas could be assigned to different stages of HPV-induced transformation. Hence, our *in vitro* model system provides a valuable tool to further functionally address the epigenetic alterations that are common in cervical carcinomas.

Worldwide carcinoma of the uterine cervix is a leading cause of cancer-related death for women (Pisani *et al*, 2002). The majority of cervical carcinomas are squamous cell carcinomas (SCCs), whereas adenocarcinomas (AdCAs) account for the remaining 15–20% of cases (Fu and Reagan, 1989; Pisani *et al*, 2002).

Development of cervical cancer is causally related to infection with high-risk human papillomaviruses (hrHPVs), predominantly types 16 and 18 (zur Hausen, 2002; Munoz *et al*, 2003). Although hrHPVs can be detected in virtually all cervical SCCs and AdCAs (Walboomers *et al*, 1999; Zielinski *et al*, 2003; Castellsague *et al*, 2006), progression of an hrHPV-positive premalignant lesion to invasive cancer is a rare event. Consistent with the multistep nature of human carcinogenesis, additive host cell alterations drive progression to invasive cancer (Snijders *et al*, 2006). Insight in these additional events may provide novel biomarkers for risk assessment of hrHPV-infected women. These events may involve chromosomal alterations affecting structure and expression of (candidate) oncogenes and tumour suppressor genes, as well as epigenetic alterations. The latter, including both histone modifications and DNA methylation, provide one mode of tumour suppressor gene silencing (Esteller and Herman, 2002). DNA methylation generally refers to the addition of a methyl group to the 5′ position of a cytosine base preceding a guanine. Methylation of CpG-rich sequences, so-called CpG islands, which are often present in gene promoter sequences, usually inhibits gene transcription.

Molecular markers based on DNA methylation, that is methylation markers, are of particular interest as recent studies indicate that DNA methylation can be easily detected in cervical scrapes using sensitive PCR-based methods like methylation-specific PCR (MSP). Moreover, positive MSP results in cervical scrapes represented methylation of respective genes in the underlying epithelium (Reesink-Peters *et al*, 2004; Feng *et al*, 2005).

To date, a number of studies have described aberrant methylation of established or candidate tumour suppressor genes in cervical carcinoma tissues, which include genes involved in apoptosis, WNT signalling, Ras-signalling and tumour invasion and metastasis (reviewed by Duenas-Gonzalez *et al*, 2005). In several of these studies, methylation of up to 16 gene promoters has been tested on cervical cancer biopsies, showing that up to 93% of them were positive for at least one of the methylation markers tested (Dong *et al*, 2001; Virmani *et al*, 2001; Narayan *et al*, 2003; Gustafson *et al*, 2004; Widschwendter *et al*, 2004; Feng *et al*, 2005; Zambrano *et al*, 2005; Wisman *et al*, 2006). However, for most of the genes studied, it is still unclear to what extent and at what stage promoter methylation reflects a functionally important step in the transformation process. This information will greatly contribute to the design of a clinically optimal marker panel for high-grade premalignant cervical lesions and cervical cancer in terms of both sensitivity and specificity.

In order to decipher functionally important steps in HPV-mediated transformation, a longitudinal *in vitro* model system of hrHPV-transfected keratinocytes (Steenbergen *et al*, 1996) can, next to cervical cancer cell lines, be of great value. We have previously transfected primary human keratinocytes with full-length HPV types 16 or 18, resulting in four immortal keratinocyte cell lines, that is FK16A and FK16B containing HPV16, and FK18A and FK18B containing HPV18 (Steenbergen *et al*, 1996). With increasing passage, these cell lines revealed increasing severity of dysplastic features in organotypic cultures that are reminiscent of the various stages of premalignant cervical lesions (Steenbergen *et al*, 1998b). Moreover, consecutive passages showed accumulation of chromosomal alterations overlapping with those found in cervical (pre)malignant lesions (Steenbergen *et al*, 1998a, 1998b, 2005; Wilting *et al*, 2006) and shared altered expression of certain genes with these (pre)cancer stages (Steenbergen *et al*, 1996, 2002; van Duin *et al*, 2003). It is currently, however, unknown to what extent this system also provides a valuable model for studying epigenetic alterations that are common in cervical carcinomas.

A novel method to assess the methylation status of multiple genes is methylation specific multiplex ligation-dependent probe amplification (MS-MLPA). MS-MLPA is a PCR-based technique allowing the semiquantitative detection of changes in DNA promoter methylation of multiple genes in a single reaction (Nygren *et al*, 2005). Discrimination between methylated and unmethylated targets is based on the annealing of probes containing a recognition site for the methylation-sensitive restriction enzyme *Hha*I.

In this study, we first assessed, by MS-MLPA, the genes out of the 29 (candidate) tumour suppressor genes that are methylated in cervical SCCs and AdCAs. Next, we determined at what stage during HPV-mediated transformation methylation of identified targets became manifest.

## MATERIALS AND METHODS

### Tissue specimens, cell lines and DNA isolation

Frozen specimens of 16 SCCs and eight AdCAs were collected during the course of routine clinical practice at the VU University Medical Center in Amsterdam. Serial cryosections were made, the outer of which were used for histomorphological assessment. All specimens included contained >70% tumour cells. The inner sections were used for DNA extraction and HPV typing as described previously (Wilting *et al*, 2006). This study followed the ethical guidelines of the Institutional Review Board of the VU University Medical Center.

Two primary keratinocyte cell lineages (EK05-1 and EK94-2) were cultured using conditions described before (Steenbergen *et al*, 1996). The cell lines FK16A, FK16B, FK18A and FK18B have been established previously by transfection of primary foreskin keratinocytes with either full-length HPV16 (FK16A and FK16B) or HPV18 (FK18A and FK18B) (Steenbergen *et al*, 1996). The cells were grown in serum-free keratinocyte growth medium (Life Technologies, Breda, The Netherlands), supplemented with bovine pituitary extract (50 *μ*g ml^−1^), epidermal growth factor (5 ng ml^−1^), penicillin (100 U ml^−1^), streptomycin (100 *μ*g ml^−1^) and L-glutamin (2 nM). Cells were harvested using 10 mM trypsin (Life Technologies).

Anchorage-independent cell clones of FK16A and FK18B were obtained by culturing late immortal cells in soft agarose, as described previously (Steenbergen *et al*, 2004), resulting in the outgrowth of a limited number of colonies. Of each cell line, a single colony was isolated, referred to as FK16ASA and FK18BSA and expanded further. Cervical cancer cell lines SiHa, HeLa and CaSki were obtained from the American Type Tissue Culture Collection (ATCC, Manassas, VA, USA). The six low passage cervical cancer cell lines 778, 808, 866, 879 and 915 were kindly provided by Professor Dr PL Stern and cultured as described previously (Brady *et al*, 1999).

From cultured cells, DNA was isolated using the QIAmp tissue kit (Qiagen, Hilden, Germany).

### Methylation specific-multiplex ligation-dependent probe amplification

The MS-MLPA was performed as published previously (Nygren *et al*, 2005), using two probe mixtures (P041A and P041B). In total, the probe mixtures contained a panel of probes specific for 29 candidate tumour suppressor genes (Table 1). Some of the genes (i.e. *APC*, *ATM*, *BRCA2*, *CHFR*, *CDH13*, *CDKN1B*, *CDKN2A*, *CDKN2B*, *ESR1*, *FHIT*, *GSTP1*, *HIC1*, *MLH1*, *PTEN*, *RARβ*, *RASSF1A*, *STK11*, *TP73*, *CADM1* (previously referred to as *TSLC1*), *VHL*) were represented by two or three probes that each recognised a different *Hha*1 restriction site in the promoter region of the respective genes. In addition, each probe mix contained control probes that lack the *Hha*1 restriction site and were used for quantification purposes.

**Figure 1:**
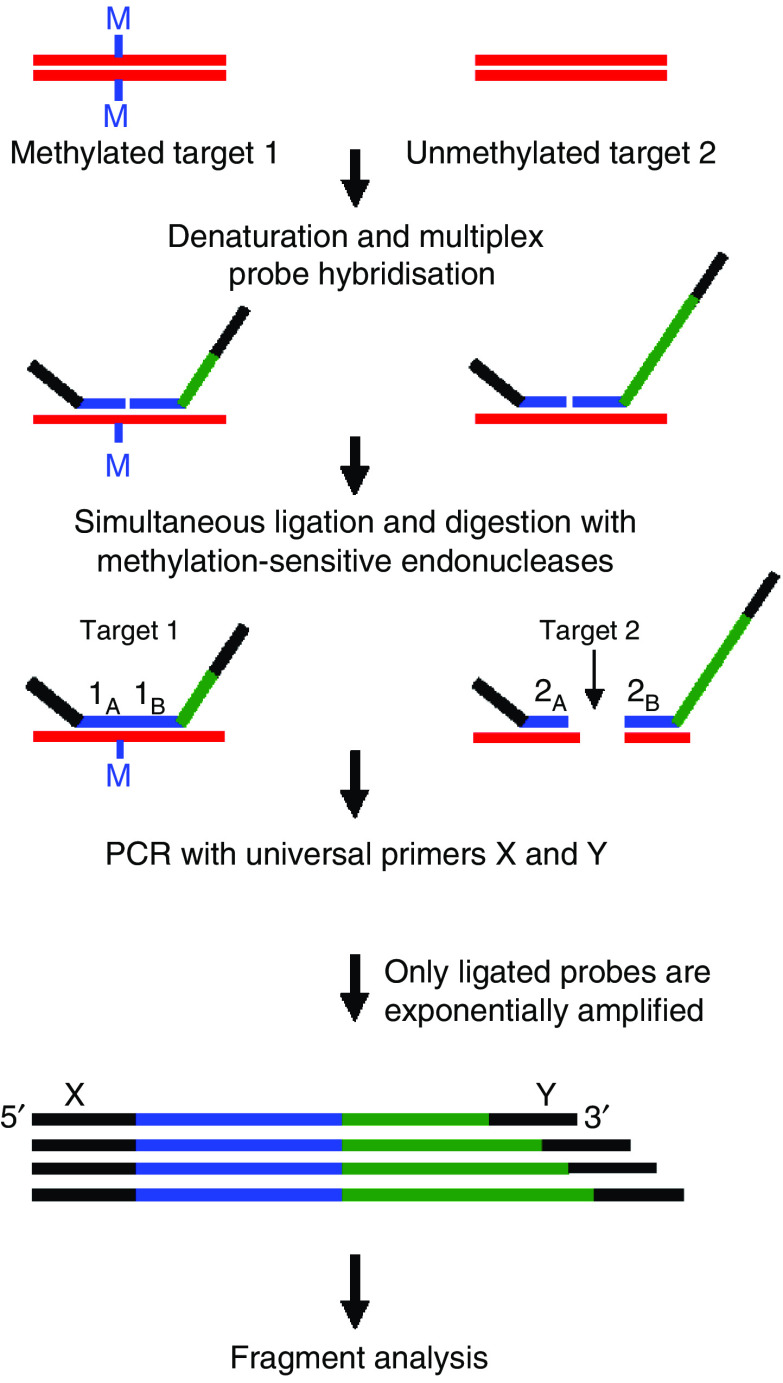
Overview of the MS-MLPA procedure (Nygren *et al*, 2005). The gene-specific probes spanning a recognition site for the restriction enzyme *Hha*1 are hybridised to the target DNA and subsequently ligated and digested with the methylation-sensitive enzyme *Hha*1. Undigested probes, that is probes of which the recognition sequence is methylated, will be amplified. If the CpG site is unmethylated, the DNA/probe complex will be digested and no amplification will take place. For each DNA sample, the MS-MLPA was performed with and without *Hha*1 digestion.

The MS-MLPA procedure is summarised in Figure 1. For each sample, 100 ng input DNA was used. After 10 min denaturation at 98°C, SALSA MLPA buffer and MS-MLPA probe mix P041A or P041B was added to the DNA, and the mixture was incubated for 1 min at 95°C, followed by hybridisation for 16 h at 60°C. After hybridisation, the samples were diluted at room temperature with H_2_O and 3 *μ*l ligase buffer A to a final volume of 20 *μ*l and divided over two series of tubes. Samples were heated to 49°C, after which a volume of 10 *μ*l of a mix containing 0.25 *μ*l ligase, 1.5 *μ*l ligase buffer B and 5 U *Hha*1 (Promega, Leiden, The Netherlands) was added. To the second series of tubes, an identical mix was added in which *Hha*1 was replaced by 50% glycerol. Ligation and digestion were performed simultaneously for 45 min at 49°C, which was followed by a 5 min incubation at 98°C to inactivate the enzymes.

To amplify the ligation products, 5 *μ*l was added to a PCR mix containing 10 × SALSA PCR buffer, 1 U SALSA polymerase and SALSA FAM PCR primer-dNTP mix (all provided by MRC Holland, Amsterdam, The Netherlands) and the following PCR protocol was used: 1 min at 95°C; 35 cycles (30 s at 95°C, 30 s at 60°C, 1 min at 72°C); 20 min at 72°C.

For fragment analysis, 8.75 *μ*l Formamide (Sigma-Aldrich, Zwijndrecht, The Netherlands), 0.25 *μ*l Gene Scan-500 ROX Size Standard (PE Applied Biosystems, Foster City, CA, USA) and 1 *μ*l PCR product was run on an ABI PRISM 3100 Avant (ABI PRISM 3100-Avant Genetic Analyzer by PE Applied Biosystems). Analysis was performed using ABI 3100 Gene Scan 3.7 software. Each amplified fragment was normalised by dividing the area under the peak by the mean of the two flanking control fragments. The percentage of methylation was calculated by dividing normalised peaks in the *Hha*1-digested reaction by the normalised peaks in the undigested control reaction. Methylation below the threshold level of 10% was considered background (Nygren *et al*, 2005). All samples were analysed twice and scored positive for methylation when in both experiments the percentage of methylation for each individual probe was above the threshold. In case of multiple probes for a single gene promoter, the gene was scored positive when ⩾1 probe showed methylation.

### Methylation-sensitive single-nucleotide primer extension (Ms-SNuPE)

Primers used to generate the specific *MGMT* PCR product for methylation-sensitive single-nucleotide primer extension (Ms-SNuPE) analysis were 5′-GTATTAGGAGGGGAGAGATT-3′ and 5′-TCTATACCTTAATTTACCAAATAACCC-3′. The PCR was performed in a total volume of 25 *μ*l containing 25 ng bisulphite-modified DNA. PCR conditions were 8 min at 95°C, 35 cycles (30 s at 95°C, 30 s at 54°C, 45 s at 72°C); 4 min at 72°C. PCR products were separated on 1% agarose gels and isolated using the GeneClean III kit (Qbiogene, Irvine, CA, USA).

The Ms-SNuPE reactions were performed mainly as described before with some modifications (Gonzalgo and Jones, 1997). Conditions for primer extension reactions were: at 95°C for 1 min, 48°C for 1 min and 72°C for 1 min. The primers used for the Ms-SNuPE analysis were 5′-GGGATTTTTATTAAGCGGG-3′ and 5′-GGGATTTTTATTAAGTGGG-3′.

The reaction was performed in a total volume of 10 *μ*l, containing 4 *μ*l of purified PCR product and 6 *μ*l PCR mix consisting of: 10 × PCR buffer (MRC-Holland), 10 pmol of each Ms-SNuPE primer, 1 *μ*Ci of either [^32^P]dCTP or [^32^P]dTTP and 1 U of *Taq* polymerase (MRC-Holland).

Stop solution was added and samples were denatured for 4 min at 95°C. Of the sample, 1.5 *μ*l was loaded on a 15% polyacrylamide gel (7 M urea).

Radioactivity was quantitated using a phosphoimager. The percentage of methylation is equivalent to the value of C/(C+T). The specimens were rated positive when methylation level reached 5%.

### Statistical analysis

Methylation percentages for individual genes in SCCs and AdCAs were compared using *χ*^2^ statistical testing. A two-sided *P-*value of ⩽0.05 was considered significant.

## RESULTS

### Promoter methylation profiles in cervical SCCs and AdCAs

**Figure 2:**
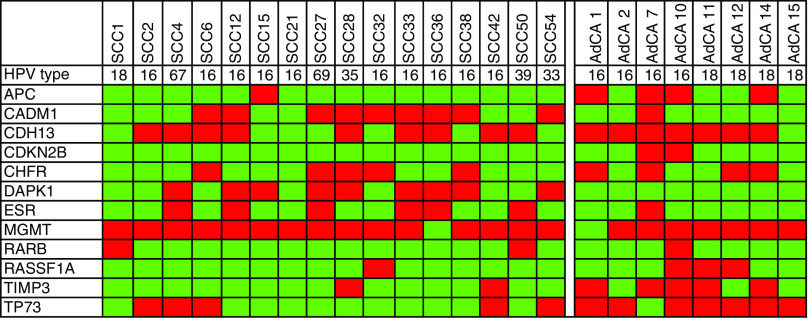
Summary of MS-MLPA results on cervical SCCs (*n*=16) and AdCAs (*n*=8). Dark boxes indicate the presence of promoter methylation; light boxes represent unmethylated CpGs. For each SCC and AdCA, the HPV type present is also shown.

To firstly identify epigenetic alterations associated with cervical cancer, we analysed promoter methylation of 29 (candidate) tumour suppressor genes by MS-MLPA in 16 SCCs and eight AdCAs. All carcinomas contained DNA of high-risk HPV types (HPV16 in 10 SCCs and four AdCAs, HPV18 in one SCC and four AdCAs, HPV33, 35 and 39 each in one SCC) or not yet classified HPV types (HPV67 and 69, each in one SCC). An overview of all genes that were methylated in the individual SCCs and AdCAs is shown in Figure 2. In addition, a summary of the overall frequencies of methylation found in SCCs and AdCAs is detailed in Table 2. Twelve of the genes showed a positive MS-MLPA result in one or more of the carcinomas tested. Squamous cell carcinomas revealed frequent promoter methylation, that is in >40% of cases, of *CDH13* (nine out of 16: 56.3%), *DAPK1* (nine out of 16: 56.3%), *MGMT* (15 out of 16: 93.8%) and *CADM1* (nine out of 16: 56.3%). In AdCAs, frequent promoter methylation of *APC* (four out of eight: 50%), *CDH13* (seven out of eight: 87.5%), *CHFR* (four out of eight: 50%), *MGMT* (seven out of eight: 87.5%), *TIMP3* (five out of eight: 62.5%) and *TP73* (seven out of eight: 87.5%) was detected.

*MGMT* was most frequently methylated in both tumour histotypes, that is in 92% of all carcinomas. On the other hand, methylation of *DAPK1* (*P*=0.007) and *CADM1* (*P*=0.04) was significantly higher in SCCs, whereas *APC* (*P*=0.01), *CDKN2B* (*P*=0.04), *RASSF1A* (*P*=0.05), *TIMP3* (*P*=0.01) and *TP73* (*P*=0.02) methylation was more common in AdCAs (Table 2).

No statistically significant different methylation profiles were found between HPV16-positive carcinomas and carcinomas containing other hrHPV types.

### Accumulation of methylation events during HPV-mediated transformation *in vitro*

**Figure 3:**
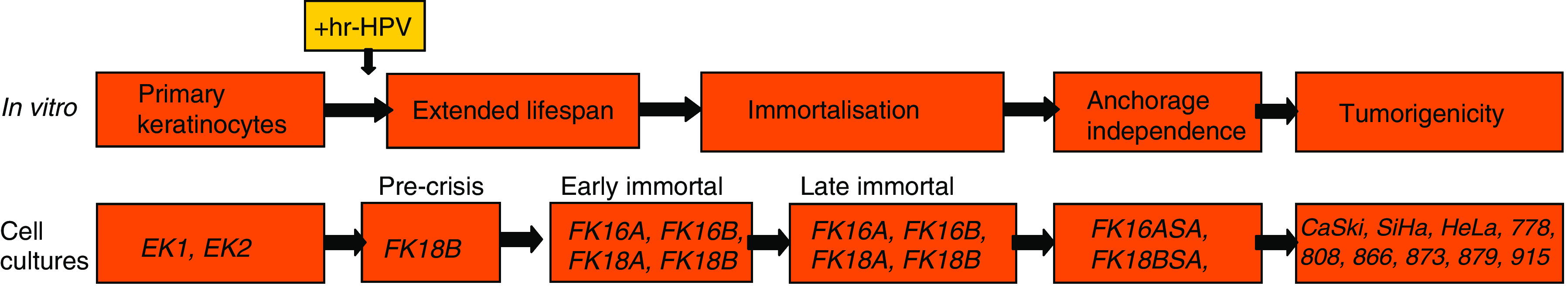
Schematic representation of the multistep process of HPV-mediated transformation *in vitro*, aligned with the hrHPV-transformed keratinocytes and cervical cancer cell lines used in this study.

To determine at which stage during HPV-mediated transformation methylation of the various genes becomes manifest, we performed MS-MLPA analysis on an *in vitro* model system of HPV-transformed keratinocytes and cervical cancer cell lines. In previous studies, we and others have shown that at least four phenotypes can be distinguished during HPV-mediated transformation: (1) extended but still finite lifespan (pre-immortal), (2) immortalisation (3) anchorage-independent growth and (4) tumorigenicity (Chen *et al*, 1993; Steenbergen *et al*, 1996, 2005). We analysed two isolates of primary keratinocytes as normal controls and one HPV18-transfected cell culture (FK18B) representing the pre-immortal stage. Moreover, early passages (passage 39–61) and late passages (passage 75–124) of two HPV16 and two HPV18 immortalised cell lines (FK16A, FK16B, FK18A and FK18B) were included, as well as anchorage-independent clones of HPV16 (FK16ASA)- and HPV18 (FK18BSA)-containing cell lines. Finally, nine hrHPV-positive cervical cancer cell lines (SiHa, HeLa, CaSki, 778, 808, 866, 873, 879 and 915), of which the latter six were tested at low passage, were included as representatives of the tumorigenic stage. A schematic representation of the cell lines analysed is shown in Figure 3.

**Figure 4:**
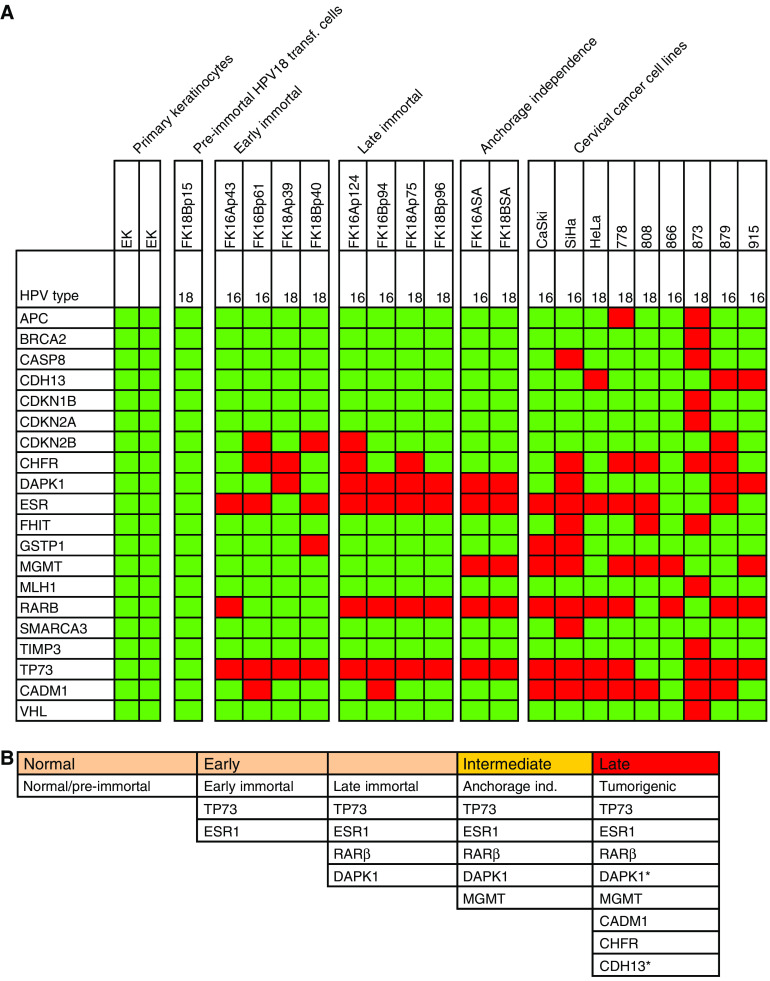
(**A**) Summary of MS-MLPA results on primary keratinocytes (EK), HPV-transformed cell lines (FK16A, FK16B, FK18A, FK18B; p=passage number) and cervical carcinoma cell lines (CaSki, SiHa, HeLa, 778, 808, 866, 873, 879, 915). Only genes (20 out of 29) that were found to be hypermethylated in at least one of the cell lines are shown. HPV types are listed for each cell line. (**B**) Longitudinal scheme of epigenetic events associated with hrHPV-mediated transformation as detected in >50% of hrHPV-transformed cell lines and cervical carcinoma cell lines. ^*^DAPK1 and *CDH13* methylation was detected in 33% of carcinoma cell lines. Dark boxes indicate methylation positive, light boxes indicate methylation negative.

None of the 29 candidate tumour suppressor genes showed promoter methylation in two cultures of primary keratinocytes, nor in the pre-immortal passage of the cell line FK18B (Figure 4). At the early immortal stage, promoter methylation of *TP73* was evident in all four cell lines and methylation of *ESR1* in FK16A, FK16B and FK18B cell lines. Additive methylation of *RARβ* and *DAPK1* became apparent at later immortal passages of all cell lines. Supplementary methylation of *MGMT* was associated with anchorage-independent growth of FK16A and FK18B. Cervical carcinoma cell lines, showed next to markers present in FK cell lines, also revealed frequent methylation of *CADM1* and *CHFR*.

In Figure 4A an overview of the MS-MLPA results on all cell lines is shown. In Figure 4B, all genes that were found to be methylated in more than 50% of all hrHPV-transformed cell lines at the various stages of progression and in cervical carcinoma cell lines are summarised. Notably, all eight genes that were frequently methylated in the HPV-transfected cell lines and cervical cancer cell lines (i.e. *TP73*, *ESR1*, *RARβ*, *DAPK1*, *MGMT*, *CADM1*, *CDH13* and *CHFR*) overlapped with those found to be methylated in the cervical carcinoma specimens.

### Confirmation of MS-MPLA results on MGMT promoter by Ms-SNuPE

To confirm MS-MLPA results, we selected the *MGMT* gene for further analysis, since this gene revealed the highest frequency of positive MS-MLPA test results in cervical carcinoma specimens. For this purpose, we used Ms-SNuPE analysis, by which methylation differences at specific CpG sites can be assessed in a quantitative manner (Gonzalgo and Jones, 1997). In this method, DNA is treated with sodium bisulphite followed by amplification of the target sequence using primers specific for bisulphite-converted DNA. The subsequent primer extension reaction utilises an internal primer which anneals to the PCR product and terminates immediately 5′ of the cytosine to be assayed, ^32^P-labelled dCTP and dTTP and *Taq* polymerase. This is followed by denaturing polyacryalamide gel electrophoresis and phosphorimage analysis to quantitate the ratio of C versus T. To confirm our MS-MLPA results, primers were specifically designed to examine the same CpG dinucleotide as analysed by MS-MLPA (nt −459 relative to the transcription start site).

**Figure 5:**
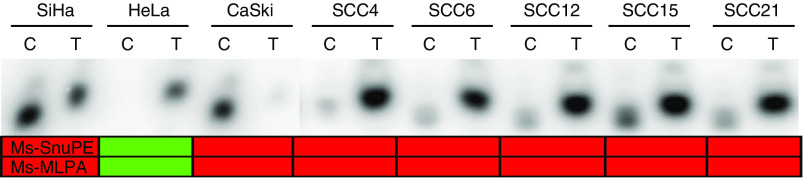
Representative Ms-SNuPE results for *MGMT* promoter methylation in a subset of cell lines and SCC specimens. Dark boxes indicate the sample was methylated and light boxes indicate the samples was unmethylated. Methylation specific-multiplex ligation-dependent probe amplification results for the same CpG dinucleotide in the *MGMT* promoter are depicted below for comparison.

Methylation-sensitive single-nucleotide primer extension analysis of all cell lines confirmed MS-MLPA results for *MGMT* in all cases (Figure 5). Cell lines SiHa and CaSki were positive in both settings while the remaining cell lines were negative for *MGMT* promoter methylation. Also in cervical cancer specimens, Ms-SNuPE results were highly comparable to MS-MLPA results (Figure 5). In all except two of the cancer specimens, *MGMT* promoter methylation revealed identical results by MS-MLPA and Ms-SNuPE. The two exceptions were SCC 42 (MS-MLPA positive, Ms-SNuPE negative) and SCC 36 (MS-MLPA negative, Ms-SNuPE positive). It should, however, be noted that in both cases percentages of methylation measured with either or both techniques were near the cutoff levels of the assays.

## DISCUSSION

Methylation specific-multiplex ligation-dependent probe amplification analysis of 29 tumour suppressor genes potentially targeted by methylation resulted in the identification of 12 methylated gene promoters in cervical carcinomas, eight of which could subsequently be associated with consecutive stages of HPV-mediated transformation *in vitro*.

The *MGMT* promoter was most frequently methylated (i.e. in 92% of carcinomas). Next to the common marker *MGMT*, also histotype-specific markers could be identified. *DAPK1* and *CADM1* were significantly more frequently methylated in SCCs while methylation of *APC*, *CDKN2B*, *RASSF1A*, *TIMP3* and *TP73* was significantly more frequent in AdCAs.

The detection of distinct methylation profiles between SCCs and AdCAs is in concordance with literature data, showing higher frequencies of *DAPK1* promoter methylation in SCCs compared with AdCAs (Dong *et al*, 2001; Narayan *et al*, 2003; Yang *et al*, 2004; Kang *et al*, 2006) and increased rates of *APC*, *RASSF1A* and *TIMP3* promoter methylation in AdCAs compared with SCCs (Dong *et al*, 2001; Narayan *et al*, 2003; Jeong *et al*, 2006; Kang *et al*, 2006; Wisman *et al*, 2006).

Based on the analysis of consecutive passages of HPV-transfected keratinocytes, we could assign methylation of the different gene promoters as detected in cervical carcinomas to distinct stages of transformation. Moreover, we showed that despite the recent finding that HPV18 E7 targets DNA methyl transferase 1 (*DNMT1*) involved in *de novo* methylation (Burgers *et al*, 2007), none of the 29 genes were found to be methylated in the pre-immortal cells expressing hrHPV E6 and E7. Only following immortalisation, an accumulation of methylated genes was detected, suggesting that inactivation of these genes is associated with a growth advantage of the hrHPV-containing keratinocytes. To what extent these genes (except *CADM1*; Steenbergen *et al*, 2004) are functionally involved in the different steps during the transformation process remains to be determined.

Methylation of both the *TP73* and *ESR1* promoter followed by *RARβ* and *DAPK1* promoter methylation were identified as rather early events associated with immortalisation. The recent demonstration of an inverse relation between *ESR1* protein expression and the severity of the cervical lesion (Bekkers *et al*, 2005) supports our observation of *ESR1* promoter methylation being a rather early event during cervical carcinogenesis.

*MGMT* promoter methylation appeared as an intermediate event, which is in line with earlier studies showing *MGMT* promoter methylation in 26% of invasive cancers and 29% of high-grade CIN lesions, compared with only 3% of low-grade CIN lesions (Virmani *et al*, 2001).

Methylation of *CHFR*, *CDH13* and *CADM1* was only observed in cervical carcinomas and cervical carcinoma cell lines and can as such be designated as relative late events. To the best of our knowledge, this is the first study showing methylation of *CHFR* to be associated with cervical carcinogenesis. *CHFR* promoter methylation has, however, been described in other types of cancer, such as breast (Tokunaga *et al*, 2006), gastric (Kang *et al*, 2004) and colorectal cancer (Brandes *et al*, 2005).

The detection of *CADM1* promoter methylation in cervical cancer cell lines is in concordance with our previous study showing reduced *CADM1* mRNA expression associated with promoter methylation in nearly all cervical cancer cell lines, and not in HPV-immortalised cells (Steenbergen *et al*, 2004).

Validation of our MS-MLPA results by Ms-SNuPE analysis, an alternative method to specifically assess methylation of a single CpG dinucleotide, confirmed the presence of *MGMT* methylation (at position −459 relative to the transcription start site), in 92% of carcinomas. The frequency of *MGMT* methylation as detected by both techniques was different from most previous studies on cervical carcinomas, in which by MSP analysis *MGMT* methylation frequencies varying from 6.7 to 38% have been reported (Dong *et al*, 2001; Virmani *et al*, 2001; Yang *et al*, 2004; Lin *et al*, 2005). Interestingly, application of the same MSP assay as described in these previous studies, by which methylation at the region from +3 to +137 relative to the transcription start site was analysed, showed evidence for methylation in only 31% of our carcinomas (data not shown). This apparent discrepancy may, at least in part, be explained by heterogeneity of *MGMT* promoter methylation in cervical carcinomas.

On the other hand, MSP analysis of the cell lines for *DAPK1*, *ESR1*, *RARβ* and *CADM1* promoter methylation showed a 78–89% concordance between the MS-MLPA and MSP results (data not shown). The absence of complete concordance may be explained by the fact that MS-MLPA is only based on a single CpG site compared to on average 4–6 CpG sites in MSP assays. Moreover, different CpG dinucleotides were targeted by MS-MLPA and MSP, indicating that, similar to *MGMT*, in the few discrepant cases heterogeneous methylation patterns may exist within the individual gene promoters.

Taken together, the present data illustrate the significance of our *in vitro* model. In our previous studies we already showed that both morphologically and genetically, the HPV-transfected cell lines closely resembled cervical (pre)malignant lesions (Steenbergen *et al*, 1996, 1998a, 2002; van Duin *et al*, 2003). The current study indicates that also with respect to DNA methylation, this model system nicely mimics cervical carcinogenesis *in vivo*. Consequently, it provides a valuable tool to analyse the functional involvement of the identified genes in the respective phenotypical alterations during HPV-induced transformation.

Next to future functional studies, the linkage of the different methylation events to distinct stages of HPV-induced malignant transformation provided first insight in the potential clinical applications of these markers. Early and intermediate methylation events may provide markers for better risk assessment of hrHPV-positive women with (ab)normal cytology. On the other hand, late methylation events or a combination of the various events may be used at the time of diagnosis for cancer staging and grading or selection and monitoring of therapy. Future clinical validation studies, for which specific guidelines have been proposed by Pepe *et al* (2001), on cervical (pre)malignant and cervical scrapes, will ultimately reveal the diagnostic value of the different methylation markers. It should be taken into account that the specificity and sensitivity of the different markers may need to be determined for individual target population as frequencies of promoter methylation can vary among different ethnic groups (Enokida *et al*, 2005; Das *et al*, 2006).

Gene alterations previously detected in our *in vitro* model system, such as *GATA-3*, *hTERT*, *MGP* and *CADM1*, were also found in clinical samples (Steenbergen *et al*, 2001, 2002, 2004; de Wilde *et al*, 2007). Therefore, we believe that the methylation events identified in the *in vitro* model system provide potential markers for cervical cancer detection as well, which is underlined by their large overlap with methylated gene promoters found in cervical carcinomas.

In conclusion, MS-MLPA has proven to be a powerful tool to identify genes targeted by DNA methylation in cervical carcinomas. In addition, we were able to link promoter methylation of eight of the 12 identified markers to distinct stages of HPV-induced malignant transformation. This resulted in more insight into the natural sequence of methylation events as well as novel candidates for future functional and clinical marker validation studies.

## Figures and Tables

**Table 1:** Genes represented by the probe mixtures (41A and 41B)

**41A**		**41B**	
TIMP3	Tissue inhibitor of metalloproteinases-3	BRCA1	Breast cancer 1
APC	Adenomatosis polyposis coli	BRCA2	Breast cancer 2
CDKN2A	Cyclin-dependent kinase inhibitor 2A; p16	ATM	Ataxia telangiectasia mutated
MLH1	mutL homologue 1	TP53	Tumour protein 53
ATM	Ataxia telangiectasia mutated	PTEN	Phosphatase and tensin homologue deleted on chromosome 10
RARB	Retinoic acid receptor, beta	SMARCA3	SWI/SNF-related, matrix-associated, actin-dependent regulator of chromatin, subfamily a3
CDKN2B	Cyclin-dependent kinase inhibitor 2B; p15	CHFR	Checkpoint with forkhead and ring finger domains
HIC1	Hypermethylated in cancer 1	CDH13	Cadherin 13
CHFR	Checkpoint with forkhead and ring finger domains	TP73	Tumour protein p73
CASP8	Caspase 8	STK11	Serine/threonine kinase 11
CDKN1B	Cyclin-dependent kinase inhibitor 1B; p27	VHL	von Hippel–Lindau
PTEN	Phosphatase and tensin homologue deleted on chromosome 10	GSTP1	Glutathione *S*-transferase pi
BRCA2	Breast cancer 2	HIC1	Hypermethylated in cancer 1
CD44	CD44 molecule	ESR1	Estrogen receptor 1
RASSF1A	RAS association domain family 1A	RB1	Retinoblastoma 1
DAPK1	Death-associated protein kinase 1	FHIT	Fragile histidine triad gene
VHL	von Hippel–Lindau	STK11	Serine/threonine kinase 11
ESR1	Estrogen receptor 1	CADM1	Cell adhesion molecule 1
RASSF1A	RAS association domain family 1A	MGMT	*O*6-methylguanine-DNA methyltransferase
TP73	Tumour protein p73	CDKN1B	Cyclin-dependent kinase inhibitor 1B; p27
FHIT	Fragile histidine triad gene	APC	Adenomatosis polyposis coli
CADM1	Cell adhesion molecule 1	CDKN2B	Cyclin-dependent kinase inhibitor 2B; p15
CDH13	Cadherin 13	CDKN2A	Cyclin-dependent kinase inhibitor 2A; p16
GSTP1	Glutathione *S*-transferase pi	RASSF1A	RAS association domain family 1A
MLH1	MutL homologue 1	RARB	Retinoic acid receptor, beta

Probes present in both probe mixtures representing the same gene recognise a different restriction site in corresponding promoter regions.

**Table 2:** Methylation frequencies in SCCs and AdCAs

**Gene**	**SCC**	**AdCa**	***P*-value**
*APC*	6.3% (1/16)	50% (4/3)	0.01
**CADM1**	56.3% (9/16)	6.25% (1/8)	0.04
CDH13	56.3% (9/16)	87.5% (7/8)	0.13
***CDKN2B***	0% (0/0)	25% (2/0)	0.04
CHFR	31.3% (5/16)	50% (4/0)	0.37
**DAPK1**	56.3% (9/16)	0% (0/0)	0.01
ESR1	37.5% (0/16)	12.5% (1/8)	0.20
MGMT	93.8%(15/16)	87.5% (7/8)	0.60
RAR*β*	12.5% (2/16)	12.5% (1/8)	1.00
***RASSF1A***	6.3% (1/16)	37.5% (3/0)	0.05
***TIMP3***	12.5% (2/16)	62.5% (5/0)	0.01
***TP73***	31.3% (5/16)	87.5% (7/0)	0.01

Genes indicated in bold italics are significantly more frequently methylated in AdCAs than SCCs, and those in bold show a significantly higher frequency of methylation in SCCs.
